# Paediatric traumatic brain injury: unique population and unique challenges

**DOI:** 10.1093/brain/awaf459

**Published:** 2025-12-13

**Authors:** Shruti Agrawal, Rebekah Mannix, Vicki Anderson, Miriam H Beauchamp, Adam Ferguson, Lucia W Braga, Shu-Ling Chong, Anthony Figaji, Christopher Giza, David K Menon, Michael J Bell

**Affiliations:** Paediatric Intensive Care, Department of Paediatrics, Cambridge University Hospitals, Cambridge CB2 0QQ, UK; Department of Paediatrics, University of Cambridge, Cambridge CB2 0QQ, UK; Division of Emergency Medicine, Harvard Medical School, Boston Children’s Hospital, Boston, MA 02115, USA; Clinical Sciences Research, Murdoch Children’s Research Institute, Mental Health Service, Royal Children’s Hospital & School of Psychological Science, University of Melbourne, Melbourne, VIC 3052, Australia; Department of Psychology, University of Montreal, Canada & CHU Sainte-Justine Azrieli Research Center, Montreal, Quebec, Canada H3T 1C5; Brain and Spinal Injury Center (BASIC), Department of Neurological Surgery, University of California San Francisco (UCSF), San Francisco Veterans Affairs Healthcare System, San Francisco, CA 94143, USA; Psychiatry and Behavioural Science, SARAH Network of Rehabilitation Hospitals, Brasilia 70000-000, Brazil; Department of Emergency Medicine, KK Women’s and Children’s Hospital, Duke-NUS Medical School, Singapore, Singapore 229 899; Division of Neurosurgery, University of Cape Town, Cape Town 7925, South Africa; Division of Paediatric Neurology, Departments of Paediatrics and Neurosurgery, UCLA Brain Injury Research Center, University of California, Los Angeles, CA 90095-6901, USA; Department of Medicine, University of Cambridge, Cambridge CB2 0QQ, UK; Department of Pediatrics, UT Southwestern, Dallas, TX 75390-9063, USA

**Keywords:** paediatrics, traumatic brain injury, evidence-based guidelines, biomarkers, neurodevelopmental outcome

## Abstract

Paediatric traumatic brain injury (pTBI) remains a leading cause of death and disability in children around the world. The evidence to support pTBI management in children notably lags that in adult populations, with a lack of data available to inform management. Injury mechanisms and physiological responses vary considerably across the developmental spectrum of childhood, bringing unique challenges to the management of pTBI. This is compounded further by complexity of neurodevelopmental changes influencing long-term outcomes.

The foundation of current understanding of pTBI is laid on the innovative work done over the turn of the century. Incremental progress in the past few years has furthered our understanding of mechanisms, disease pathophysiology, recovery pathways and consequences of pTBI. There are developments in identification of biomarkers that can help in diagnosis and predict outcomes more accurately to guide clinical decision-making and track long-term outcomes. However, this progress has been slow, and more work is required to translate the large body of observational work into interventions to help improve outcomes of pTBI.

This review aims to synthesize recent findings, evaluate existing evidence and propose future research directions. Structured initially to address key epidemiological and pathophysiological differences in the paediatric population with associated clinical challenges, followed by the potential role of physiological, blood and imaging biomarkers, this review seeks to provide a comprehensive update. Additionally, it addresses current evidence gaps in therapeutic strategies, rehabilitation needs and comprehensive systems of care, integrating insights from high- and low-resource settings. Finally, it reviews current research with a view to offering recommendations to reduce the evidence gaps in pTBI.

## Teaser

Paediatric traumatic brain injury is a leading cause of disability worldwide, yet clinical evidence to guide treatment still lags behind that for adults. Agrawal *et al.* review recent advances in understanding and management of these injuries, and outline research priorities to translate progress into better clinical outcomes.

## Introduction

According to the Global Burden of Disease Collaborative Network report, >4 million children around the world suffered a traumatic brain injury (TBI) in 2021.^1^ This high prevalence, combined with complex developmental implications and substantial disability adjusted life years (DALY) lost, underscores the urgency of enhancing both clinical management and research efforts. Although internationally relevant, evidence-based, comprehensive guidelines in paediatric TBI (pTBI) are limited, there is growing global recognition to strengthen research in this vulnerable population.^2,3^ Most current recommendations, including for those most seriously affected (children with severe TBI) are based almost entirely on level III evidence.^4-7^ The evidence gap is exacerbated further in low- and middle-income countries (LMICs), which carry the greatest disease burden and DALY, particularly for the lowest socio-demographic index (SDI).^8,9^ These regions often lack not only clinical resources, but also the infrastructure to generate and use context-appropriate data. Addressing these disparities and implementation gap is essential for equitable care globally.

The recent publication of a comprehensive, evidence-based classification system for TBI by National Institute of Neurologic Disorders and Stroke (NINDS) marks a significant milestone in advancing clinical care and research in the field.^10^ This framework provides recommendations covering six key domains: clinical/symptoms; imaging; blood-based biomarkers; psychosocial and environmental modifiers; implementation science; and retrospective classification. However, persistent gaps in pTBI evidence across all the domains precluded the development of specific recommendations for children within this new classification. Owing to significant physiological and developmental differences between adults and children, an age-appropriate approach to assessment and management is essential, thereby limiting the applicability of adult-based recommendations to pTBI. In addition, social determinants of health play a crucial role in influencing outcomes and must be incorporated in pTBI clinical care and research framework. High-quality paediatric-specific evidence is therefore urgently needed across the full spectrum of pTBI severity (mild, moderate and severe) to build comprehensive, developmentally informed clinical guidelines that reflect global realities. Scientific advances in clinical trial designs, biomarker discovery, neuroimaging, systems-based care models and longitudinal outcome-tracking offer possibilities of fulfilling this goal.

This review, therefore, evaluates existing evidence, identifies gaps and proposes future research directions for the field of pTBI. Relevant papers were identified through a PubMed search using various combinations of keywords (Supplementary material, Methods). With a view to synthesizing current evidence, articles published in the past 5 years were prioritized, and key early research was incorporated for context and reinforcing concepts where necessary. Although not exhaustive, by explicitly addressing the full spectrum of pTBI severity and highlighting disparities in care and evidence generation, it provides a forward-looking foundation for improving pTBI outcomes globally. Structured around epidemiology and developmental considerations, followed by diagnosis, management and outcomes, and finally, the systems of care and prevention, this review offers an updated and integrated perspective on pTBI. It incorporates evidence and implementation challenges from both high-income and resource-limited settings to inform globally relevant strategies and provides recommendations to advance research in pTBI.

## Epidemiology

Key takeaway: the true incidence and prevalence of pTBI are likely to be vastly underestimated, particularly in low-resource countries, owing to underreporting and lack of formal care. This issue is further complicated by incomplete mortality data, regional variations in injury definitions and documentation, and limited healthcare access.

**Figure 1:**
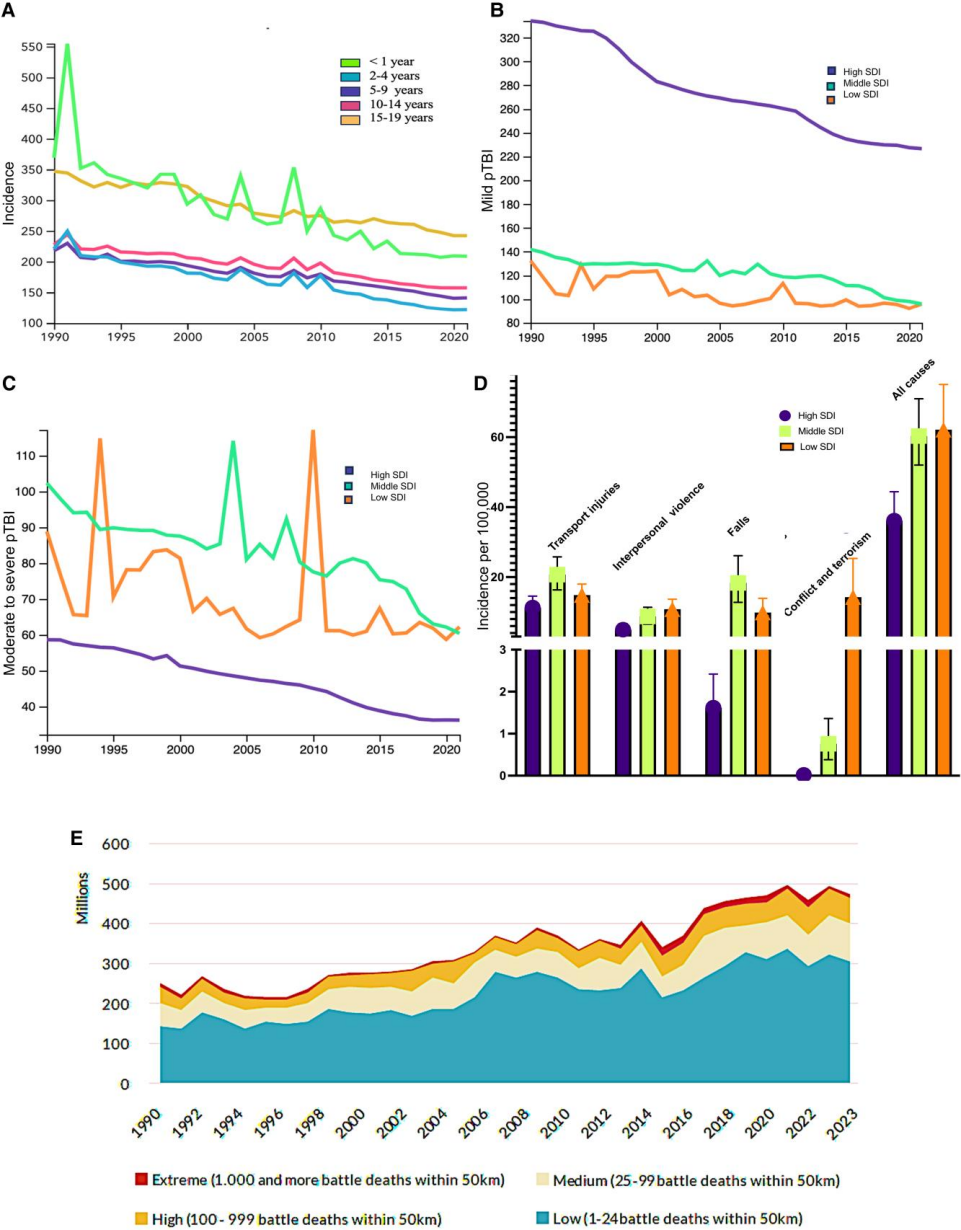
**Global trends in paediatric traumatic brain injury (pTBI).** (**A**–**D**) Incidence of pTBI trends by (**A**) age group, by socio-demographic index (SDI) for mild pTBI (**B**) and moderate to severe pTBI (**C**), and by mechanisms of injury (**D**), using data from the Global Burden of Disease Collaborative Network Study 2021 (Institute for Health Metrics and Evaluation, Seattle, WA, USA, 2024).^1^ (**E**) Temporal trends in the number of children in conflict-affected zones by conflict-zone intensity from 1990 to 2023, reproduced with permission, from the Peace Research Institute Oslo, PRIO. Originally created by Østby, Gudrun and Siri Aas Rustad for Save the Children, Conflict Trends 1, https://www.prio.org/publications/14180.^13^

The worldwide incidence of pTBI varies greatly by country and region. Historical estimates suggest 47–280 pTBI per 100 000 children.^11^ However, this is likely to be vastly underestimated, owing to lack of standardized, international reporting infrastructure, particularly in LMICs.^12^ About 80% of pTBI cases are defined as concussion or mild TBI, characterized by a Glasgow Coma Scale score of ≥13 and no abnormal findings on conventional structural imaging (Fig. 1A).^11^ Given that many mild TBI go unreported, estimating the true incidence of pTBI based solely on hospital admissions or emergency department visits might underestimate the real numbers by as much as 8- to 9-fold.^14^ Several population-based studies from high-income countries (HICs) suggest a 20%–30% prevalence of mild pTBI.^15-18^

A bimodal age distribution of pTBI incidence is often described, with peaks in children <2 years of age and in adolescence (Fig. 1B).^19^ Males have ∼1.5 times higher rates of pTBI than females.^11^ Falls, transport and sports-related injuries, firearm injuries and interpersonal violence account for most injury causes, although these data vary by SDI (Fig. 1C).^11,20,21^ Most pTBI-related deaths occur largely owing to road traffic events, interpersonal violence, conflict and terrorism (Fig. 1C).^1^ In Africa and Asia, pedestrians are most frequently injured in motor vehicle collisions, whereas vehicle occupants are more commonly affected in Australia, Europe and the USA.^22^ Children in low-SDI countries face >500-fold increased risk of sustaining a TBI attributable to conflict and terrorism, predisposing them to additional risks from blasts, firearm injuries and abuse-related head trauma (Fig. 1D).^1,13,23^ In 2022, ∼468 million children (18.8%, or more than one in six) were living in a conflict zone, and pTBI in war zones are a severe consequence of armed conflict. Blast injures are the most common mechanism (57%), followed by gunshots (29%) and fragments (14%). The average age of affected children is 8–10 years, with both male and female children affected, although cultural factors often lead to higher reporting of male injury. Mortality rates vary significantly, ranging from 3% to 47% depending on the severity of the conflict, injury and access to healthcare. There is an urgent need for more research, training and specialist deployment in conflict areas to improve outcomes for pTBI cases in war zones.

**Figure 2:**
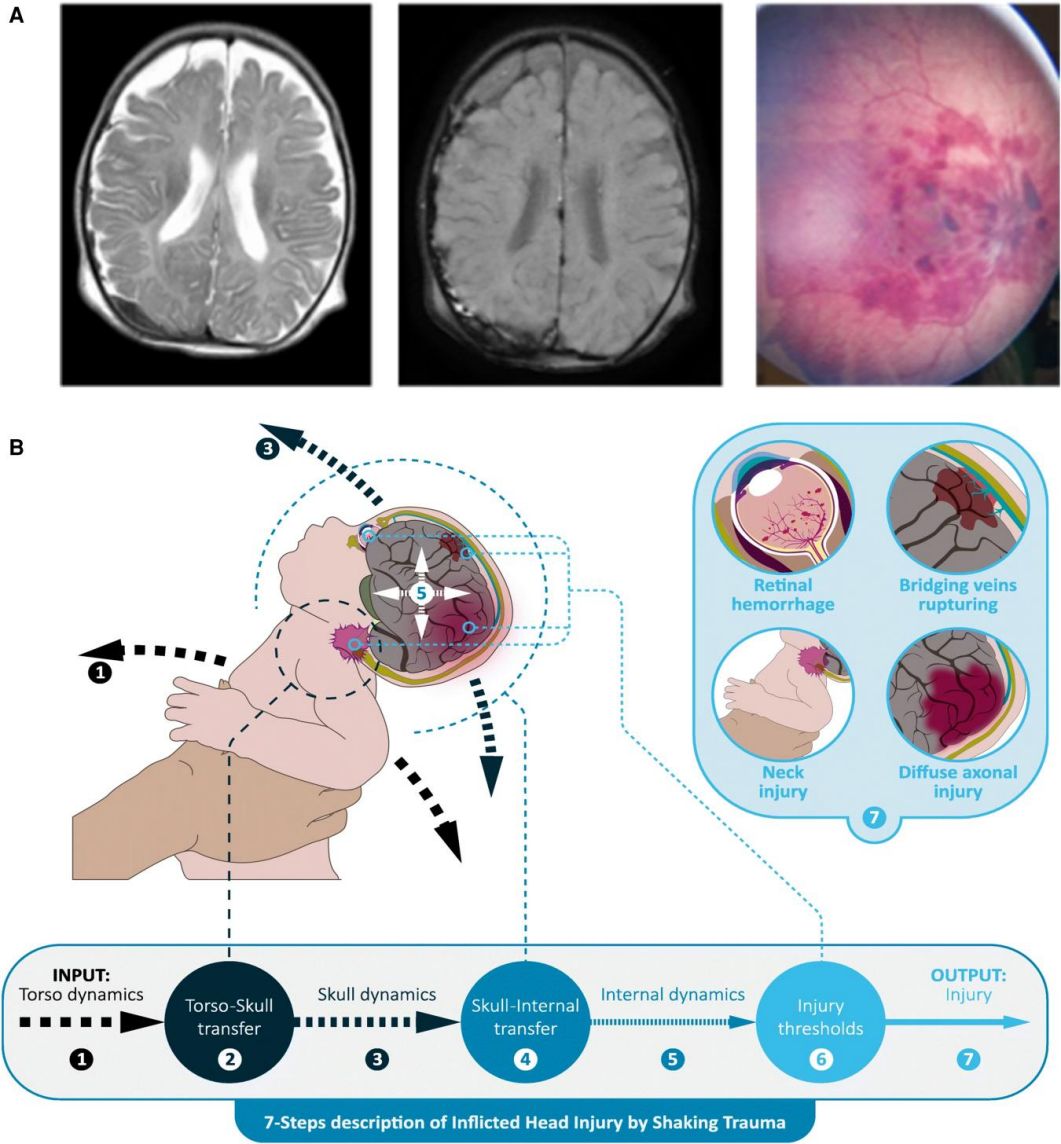
**Abusive head trauma (AHT) in children.** (**A**) Neuroimaging and fundoscopic findings in AHT: subdural haematoma (*left* and *middle*) and retinal haemorrhages (*right*). (**B**) Biomechanical modelling of AHT by shaking trauma in children showing force transfer, skull and internal dynamics, and resulting injury patterns (adapted from van Zandwijk *et al*.,^26^ under CC BY 4.0).^26^

Abusive head trauma (AHT) is an injury unique to infants and toddlers for which: (i) timely recognition is often delayed because of the relationship of the perpetrator to the infant; (ii) patterns of injury leading to severe cerebral oedema, hemispheric infarctions and greater ischaemic burden are unique; and (iii) outcomes are worse in comparison to accidental pTBI (Fig. 2).^24-29^ The global incidence of AHT is difficult to assess given the nature of the injury, compounded further by lack of legislation and underreporting in many parts of the world. Estimates suggest that AHT could account for 25%–50% of pTBI in young children, and data from HICs suggest an incidence of 25–35 per 100 000 infants per year (Supplementary Box 1).^27^

Hospital mortality rates for pTBI are >10-fold higher in LMICs (3%–10%)^30,31^ in comparison to HICs (0.1%–0.4%).^32,33^ Several factors (limited pre-hospital care, delayed access to tertiary services and inadequate acute management infrastructure) are likely to contribute to this alarming disparity.

Despite some recent encouraging global reductions in rates of pTBI, substantial inequities persist (Fig. 1). As highlighted above, LMICs bear the major brunt of the disease burden. Lack of standardized international reporting and trauma registries limits the global understanding of the true burden of pTBI. To strengthen epidemiological understanding, consistent case definitions, international data-sharing mechanisms and region-specific surveillance strategies are required.

## Developmental considerations

Key takeaway: understanding the age-specific neurodevelopmental impact on injury and recovery requires developmentally appropriate assessment tools across the paediatric age span, along with integration of family and environmental influences. Comprehensive insights into long-term outcomes and effective interventions require consideration of the parallel and dynamic nature of developmental and recovery processes.

**Figure 3:**
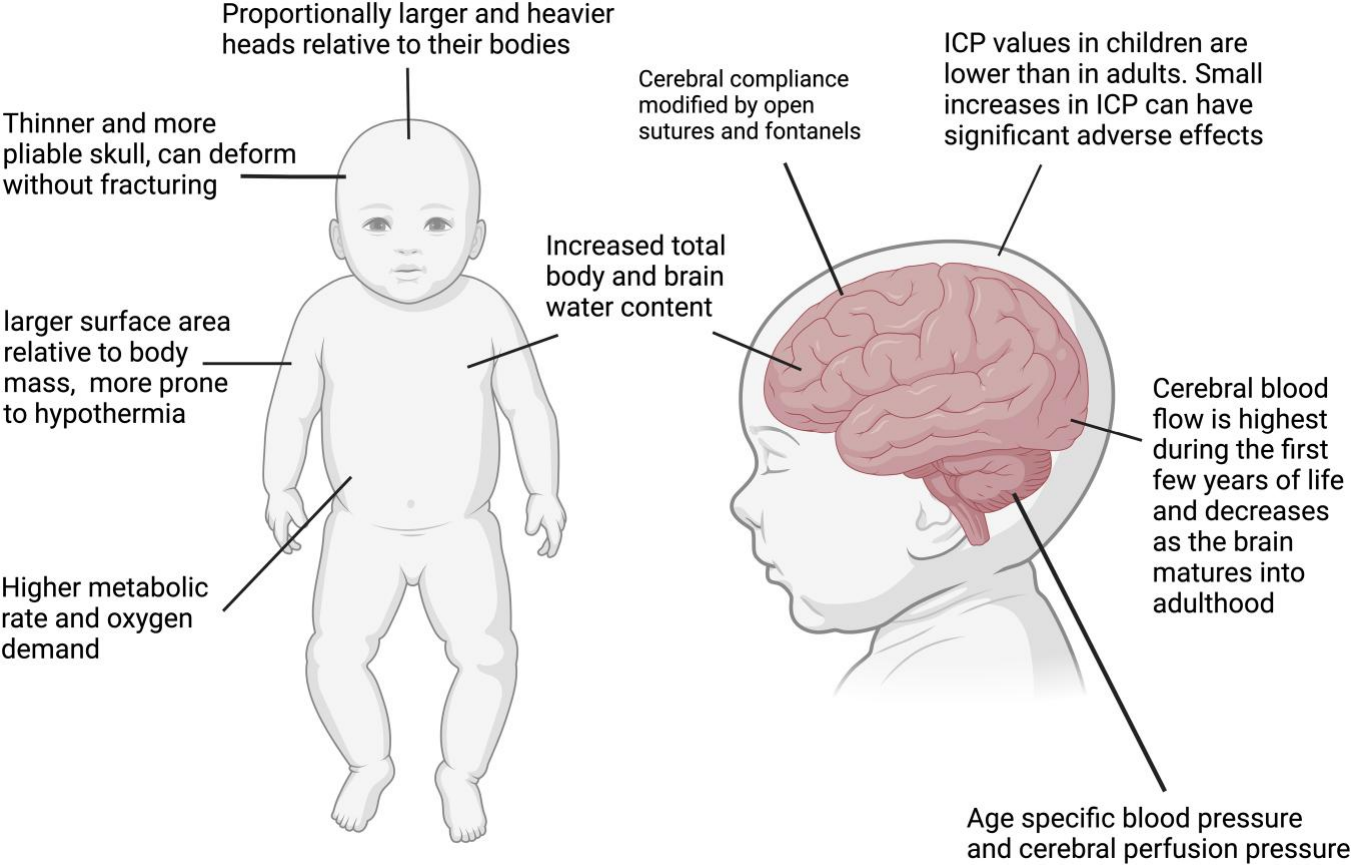
**Unique physiology of paediatric traumatic brain injury.** Created in BioRender. Mannix, R, 2025, https://BioRender.com/6q2o9fj. ICP = intracranial pressure.

The anatomy and physiology of children differ markedly from adults (Fig. 3).^34^ There are systemic and brain-specific developmental differences across childhood. These differences span multiple domains (including anatomical, physiological, biochemical, cellular and molecular changes) that influence not only the pathophysiology of pTBI but also the recovery trajectory and long-term outcomes. Although a detailed exploration of all the differences is beyond the scope of this article, several recent reviews provide a comprehensive overview of these developmental factors.^35^ The concepts discussed here align with the goal of this review of synthesizing clinically relevant insights that inform assessment and management.

### Anatomical and physiological differences

Proportionately larger head size in children, combined with underdeveloped neck muscles and ligaments, leads to unique biomechanical susceptibilities to rotational and acceleration–deceleration forces during traumatic events. Additionally, the bony elements remain unfused for ≤15 months after birth, which contributes to age-related changes in cerebral compliance. The brain contains 15%–20% more water early in development. Combined with immature axonal structures and incomplete myelination, this makes children more susceptible to diffuse injuries.^36^

To meet the high metabolic demands, cerebral blood flow (CBF) increases rapidly in the first year of life and continues to rise throughout early childhood. From mid-childhood, CBF gradually slows towards adult levels.^35^ Developmentally dependent differences in cerebral autoregulatory mechanisms, cerebral haemodynamics and cerebral metabolism throughout childhood have specific injury-related implications for CBF.^37^ These changes influence the susceptibility of the paediatric brain to perfusion disturbances, formation of oedema^38^ and metabolic insults, such as glucopenia.^39^

Neurotransmitter systems, particularly GABAergic and glutamatergic pathways, are still maturing, which results in elevated NMDA receptor activity.^38^ The vulnerability of the immature brain to oxidative stress and excitotoxicity is driven by excessive glutamate release, reduced glutathione peroxidase capacity, immature inhibitory neurotransmission and underdeveloped mechanisms for ion homeostasis. This leads to a high incidence of post-traumatic seizures and status epilepticus after pTBI in younger children.^40,41^ Preclinical models and adult data suggest a high incidence of spreading depolarization characterized by waves of near-complete neuronal and glial depolarization that propagate across the cerebral cortex.^42^ These events can exacerbate secondary brain injury by disrupting ionic gradients, impairing mitochondrial function and causing local reductions in CBF. Although there are no paediatric data available on spreading depolarizations, the increased vulnerability of a young brain to excitotoxicity suggests that spreading depolarizations might hold therapeutic promise in pTBI.

### Neurodevelopmental differences

Rapid neuronal and glial development, synaptogenesis and pruning, and ongoing myelination in the first years of life confer both increased vulnerability and the potential for plasticity and adaptability.^38^ Injury during these sensitive periods, when cognitive, social and behavioural functions are still emerging and unconsolidated, can disrupt the acquisition of crucial skills and maturation of functional neural networks.^43^ These disruptions contribute to the persistent cognitive and behavioural difficulties seen after pTBI.^44^ Neuroplasticity of the developing brain also allows the ability to reorganize and support compensatory mechanisms after injury. However, this can be maladaptive, such as crowding effects or delayed deficits that emerge later in development.^44^ The interaction between vulnerability and plasticity is a complex interplay between age at injury, injury severity and ongoing development.^44^ Understanding this balance is essential for interpretation of the neurobiological mechanisms and metabolic consequences of pTBI. Children also have fewer lifetime co-morbidities, such as hypertension or age-related vascular/metabolic impairments, in comparison to adults. This physiological advantage, combined with heightened neuroplasticity, provides an opportunity to develop longitudinal rehabilitation strategies that could improve functional outcomes in children and warrants further exploration.^38,45^

**Figure 4:**
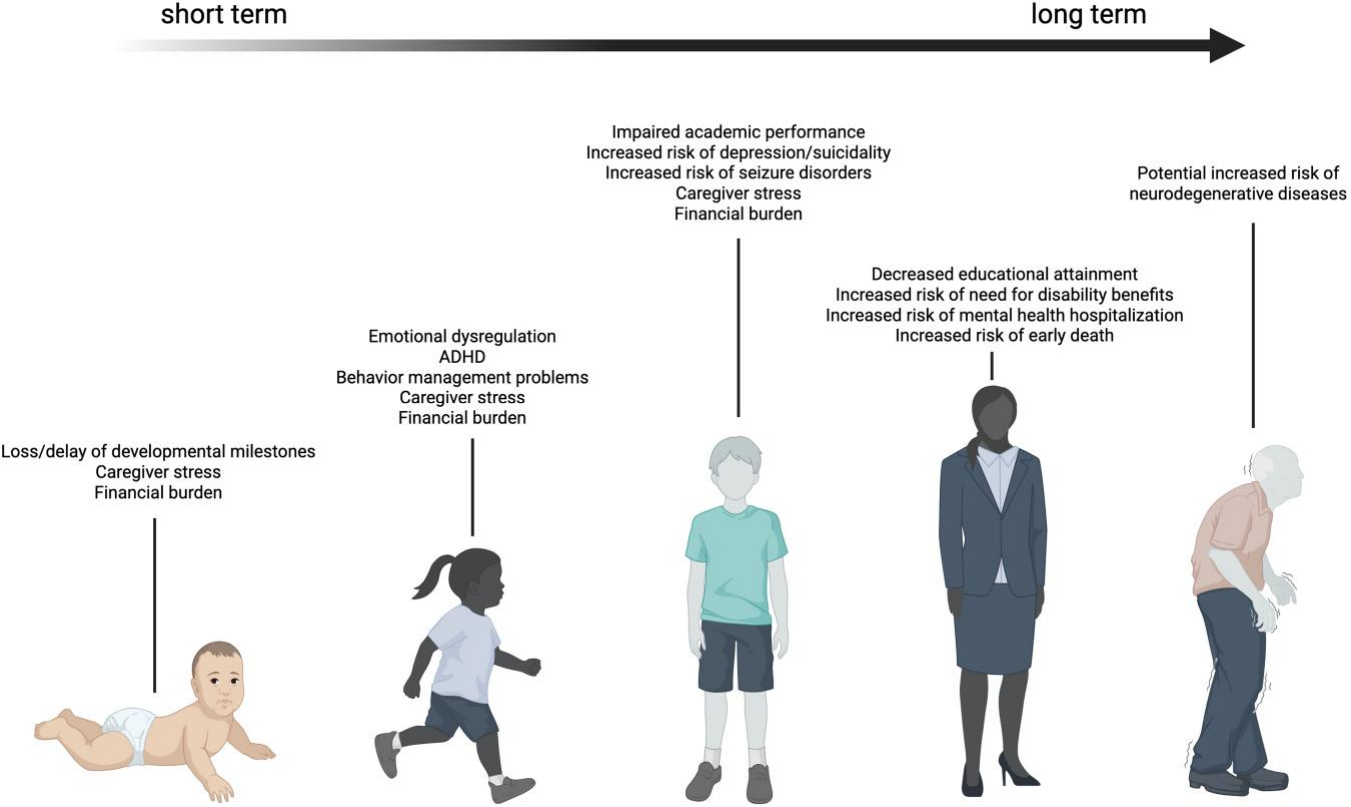
**The burden of paediatric traumatic brain injury across the lifespan.** Created in Biorender: Mannix, R, 2025, https://BioRender.com/f5zzgk3. ADHD = attention deficit hyperactivity disorder.

Beyond cognitive impairments, pTBI also increases the risk of secondary neurodevelopmental and psychiatric diagnoses, including autism spectrum disorder, attention deficit hyperactivity disorder and mood or anxiety disorders (Fig. 4).^46-48^ These conditions may emerge or persist across the lifespan, further amplifying the long-term burden of pTBI. Even mild injuries can cause changes in the structure or function of the brain. However, the long-term impact of pTBI on neurodegeneration is poorly understood, for which longitudinal follow-up and serial neuroimaging studies are required.^49,50^ Developmental characteristics must guide clinical care and research design. These have practical implications for age-appropriate modifications to diagnosis, treatment and rehabilitation after pTBI. Dynamic, non-linear brain maturation and cognitive development, in addition to considerable interindividual differences across the paediatric age span, bring additional complexity, complicating the interpretation of outcomes based solely on chronological age. Despite this, few studies, including large-scale observational cohorts, span the full paediatric range, and the youngest children are often excluded.^51^

### Adolescence-specific differences

Adolescence represents another unique developmental phase with ongoing reorganization of neural networks. This is marked by a linear increase in cortical white matter with a concurrent reduction in grey matter, which culminates in the frontal lobe.^52^ These neurobiological shifts contribute not only to increased risk-taking behaviour, predisposing adolescents to TBI, but also to long-term developmental impact from disruption of important maturational processes by TBI.^53,54^ Extensive hormonal changes during this period also establish sex-specific neurodevelopmental pathways. Although this offers the possibility of hormone-based treatment, pTBI-related hormonal disturbances can adversely affect the overall growth, puberty and neurodevelopmental outcomes of a child.^55^ Prevalence rates of post-traumatic hypopituitarism reach as high as 58%; growth hormone deficiency is the commonest abnormality and has a documented impact on cognitive function.^56^ Therefore, prospective, long-term studies are warranted to characterize its clinical consequences and evaluate improvements with timely diagnosis and treatment.^56^

## Diagnosis and management of paediatric traumatic brain injury

Key takeaway: robust assessment tools are essential for accurate diagnosis and evaluation of pTBI. Such tools must be developmentally appropriate and account for the complex interplay of injury, development, environment and child-specific factors. Tailored, longitudinal assessment tools, together with a biopsychosocial approach, are required to help management and guide rehabilitation.

For effective diagnosis and management of pTBI, a comprehensive, age-sensitive approach, tailored to the developmental stage, injury severity, and available healthcare resources, is essential. This section outlines the best practices for acute assessment, imaging, biomarker utilization, monitoring and rehabilitation, while distinguishing recommendations suitable for LMICs and HICs.

### Assessment and diagnosis

#### Acute assessment

The physical examination remains the mainstay of assessment in pTBI. Traditionally, the severity of TBI is defined by its effects on neurological function, mainly consciousness. Age-appropriate tools for post-concussion assessment in children are available, such as the Sports Concussion Assessment Tool (SCAT).^2^ However, validated age-appropriate clinical tools to assess neurological function (e.g. age-appropriate Glasgow Coma Scale, post-traumatic amnesia measures) are limited, which affect symptom reporting and hinder diagnosis and treatment.^57-60^ Physiological biomarkers, such as pupillary light reflex assessment, may offer critical diagnostic and prognostic information. Changes such as new asymmetry or a non-reactive pupil can signal serious conditions, such as uncal herniation. Ongoing myelination may impact the expected pupillary responses in infants, necessitating cautious interpretation.^61^ Because manual pupillary examinations can lack consistency, especially across providers, quantitative pupillometry is recommended to provide objective, reliable data on pupillary reactivity.^62,63^

CT scanning remains the primary imaging modality for acute assessment. Evidence-based algorithms and decision rules help to identify children most likely to benefit from an urgent CT scan and are especially useful in mild pTBI, where advanced imaging might not be necessary.^64-68^ Available algorithms improve diagnostic accuracy and minimize radiation risks. Developed and validated in HICs, these tools share overlapping predictors but differ significantly in their emphasis on safety and resource utilization, performance metrics, clinical impact and practical implementation.^6^ For optimal use in resource-limited settings, adopting a decision tool with simplified criteria that focus on high-yield predictors (e.g. altered consciousness, vomiting, suspected skull fracture) can help to align practice with local priorities and resources.^69^

When deemed necessary, imaging findings help to identify patients who would benefit from surgical interventions and/or neuroprotective measures in intensive care. In view of the radiation risks associated with CT in the developing brain,^70,71^ some centres are also exploring the use of fast MRI sequences for the acute evaluation of pTBI.^72^ Although MRI might miss isolated skull fractures and subarachnoid haemorrhage,^73^ it can be crucial in identifying injuries missed on CT scanning, which might have a major impact on outcome.^74,75^ From a research perspective, the current CT-based injury severity scoring systems, such as the Marshall scale or Rotterdam score, have been validated only in the adult TBI population.^76,77^

In patients who do not require neuroimaging, a comprehensive neurological examination can yield important diagnostic and prognostic information. Using standardized, objective assessments in the emergency department or shortly after injury, such as targeted visio-vestibular examinations, might improve prognostic accuracy.^78^ Clinical signs such as vestibulo-ocular dysfunction, dysdiadochokinesia and abnormal Romberg tests are often seen in mild TBI; however, their sensitivity, specificity, reliability and overall validity needs thorough evaluation in children.^79^

#### Ongoing assessment

Serial ongoing assessment is crucial to facilitate early recognition of symptoms, neurological deterioration and timely intervention to optimize recovery for any severity of pTBI. For children with mild TBI, a brief period of observation is warranted to watch for any deterioration before discharge home. For more severely injured children, serial clinical assessment should continue throughout hospitalization to prompt the need for neuroimaging or therapeutic escalation. For patients with significant impact on consciousness and/or those requiring neuroprotective measures, invasive or non-invasive monitoring is used as a surrogate for clinical neurological assessment. The Brain Trauma Foundation guidelines support the use of intracranial pressure (ICP) monitoring, whereas the advanced monitoring modalities, such as brain tissue oxygenation and assessment of cerebral autoregulation, are less established in children owing to limited normative data.^4^ Once the child is medically stable, functional assessments, such as post-traumatic amnesia assessment and physiotherapy, are warranted.

#### Chronic assessment

Comprehensive assessment covering multiple neurodevelopmental domains is required for holistic evaluation of the patients using age-appropriate assessment tools. The premorbid abilities of the child, psychosocial context, cultural background and functional status are crucial in identifying and supporting family needs and psychological status. Longitudinal assessments in pTBI are further complicated by the need to assess the age and developmental stage of the child at all time points after injury.^80-88^

### Physiological, fluid and imaging biomarkers

Key takeaway: the application of biomarkers in pTBI holds promise for linking clinical presentations to underlying pathobiology. However, it is complicated by age-dependent physiological variations, limited research on specific biomarkers for children, and challenges in adapting adult diagnostic and prognostic frameworks to paediatric contexts.

Biomarkers might help to link clinical presentations to underlying pathobiology and are part of a new approach to TBI characterization. Their use in children requires important additional considerations.

#### Physiological biomarkers (physiome)

The profound anatomico-physiological changes across childhood affect the interpretation of normal and pathological values. For example, baseline cerebral blood volume increases sharply in the first 3 years of life, and relative changes in response to stimulation are substantially greater than in adults.^89^ Although the normal ICP in children is lower, we have extrapolated adult guidelines for a single treatment threshold.^90^ Likewise, blood pressure nomograms vary with age, and age-appropriate cerebral perfusion pressure targets are unknown. Pressure autoregulation is often impaired, and its physiological implications might manifest differently in children.^91^ The use of non-invasive monitoring modalities (transcranial Doppler and near-infrared spectroscopy) is gaining popularity for their ease of use at the bedside and derived real-time information, especially in LMICs, where the availability of invasive monitoring is limited.^92-94^ Practice recommendations for clinical use of transcranial Doppler in paediatrics have helped with practical implementation.^95^ However, the underlying normative physiological and pathological thresholds for both non-invasive and invasive advanced clinical neuromonitoring modalities are not well established in children.

#### Blood and fluid biomarkers

Research on biomarkers in pTBI is growing for diagnostic and prognostic purposes. In unique pTBI settings, particularly suspected AHT, biomarkers may enhance diagnosis.^96^ Blood-based biomarkers may help to identify acute pathobiology and injury severity in moderate to severe pTBI. In milder disease, where physical examination might be normal, biomarkers can help to delineate children who would benefit from neuroimaging.^97-100^ Various biomarkers, such as GFAP (glial fibrillary acid protein), NfL (neuro-filament light chain), UCHL1 (ubiquitin C-terminal hydrolase-L1), s100b (S100 calcium binding protein B) and p-tau, can be increased in pTBI in comparison to healthy controls.^99-103^ Others, such as NSE (neuron specific enolase) and sNCAM (soluble neural cell adhesion molecule), have shown promise as early predictors of long-term attention and executive function problems. Although GFAP and UCHL1 are well established in adult mild TBI, evidence defining their utility in pTBI, in addition to age-related normative values, is still emerging.^104^ The ongoing prospective multicentre pTBI study (BRAINI-2) is expected to generate evidence to support their clinical application in children.^105^

Furthermore, specific age-dependent expression of proteins may affect both the presence and the concentration of select blood-based molecules.^101,106,107^ In children, practical considerations include limitation of invasive sampling methods, such as venepuncture and serial sampling, particularly in younger children with small circulating blood volumes. These could be circumvented by using capillary whole-blood collection or salivary microRNAs. CSF biomarkers might be more sensitive and specific but are limited to cases requiring invasive procedures.^97^ Our understanding of genomic heterogeneity in explaining variability in disease pathophysiology, treatment response and outcomes following TBI is evolving.^108,109^ In particular, recent studies have explored the association of *APOE* polymorphisms in brain-derived neurotrophic factor gene with outcomes following pTBI.^110,111^

#### Imaging biomarkers

Neuroimaging reflects developmental processes, including synaptic pruning, myelination and CBF changes.^112^ These biological processes can affect structural imaging, diffusion tensor imaging, functional connectivity and CBF measurement.^90^ Although imaging biomarkers provide high regional resolution, their use over time is limited by cost and by the need for sedation in younger children. Where resources permit, rapid sequence MRI is being explored.^72,73^ MRI also provides additional information that helps in disease characterization and prognostication. More specifically, microhaemorrhages, diffuse axonal injury and its grade detected by MRI add valuable information to understand the trajectory of recovery and rehabilitation strategies.^74,113,114^ Advanced MRI modalities hold promise in prognostic evaluation, including susceptibility weighting (sensitive to microhaemorrhages and vascular injuries), diffusion tensor imaging (white matter integrity, which can have a profound impact on long-term cognitive outcomes), diffusion-weighted imaging (assessment of oedema and ischaemia) and magnetic resonance spectroscopy (biochemical details of brain metabolism, such as *N*-acetylaspartate, choline and creatine, which influence recovery potential).^114-116^ There are encouraging results from pooled analysis of diffusion MRI data from pTBI cohorts showing altered white matter diffusion metrics in relationship to injury severity,^117^ sex-based differences in fractional anisotropy,^118^ and cerebellar atrophy.^119^ In patients with persistent symptoms, distinguishing features have been identified in diffusion MRI^120-122^ and on susceptibility-weighted imaging.^123^ Although there are advances in understanding multimodal MRI in pTBI, considerable gaps persist. Further evidence from larger, longitudinal studies is needed before specific guidance or recommendations can be established.

#### Theragnostics and endotypes

Biomarkers can guide therapeutic interventions or predict chronic sequelae (such as post-traumatic epilepsy or persisting post-concussion symptoms). Combinations of clinical features and multiple biomarkers that outline a specific pathophysiology are referred to as endotypes. As in oncology, identifying pTBI endotypes can lead to enriched cohorts, establish biological markers of treatment efficacy and improve clinical trial designs.^124^

## Clinical management of paediatric traumatic brain injury

Key takeaway: optimal outcomes for pTBI require early, tiered management tailored to injury severity, robust adherence to evidence-based guidelines (despite limited scientific support in some areas), and comprehensive, multidisciplinary rehabilitation to address long-term deficits. Limited resources and a weak evidence base, especially in LMICs, present challenges to consistent care delivery and recovery support.

In the past 5 years, many clinical practice guidelines have been published for management of different severities and phases of pTBI, mainly from HICs. However, their practical application and adherence have been limited owing to lack of robust scientific evidence, with the majority of recommendations being based on level III evidence, in addition to lack of resources in LMICs.^125^ In this section, we will discuss and summarize available scientific evidence for clinical management.

The early management is focused on limiting brain damage from the primary injury and preventing secondary insults. This is followed by rehabilitation to improve long-term outcomes. Broadly, robust pre-hospital care is required, followed by treatment at facilities with resources to manage such injuries for best outcomes.^66^ In the acute phase, only a small proportion of patients with mild TBI require hospital admission and treatment. However, children with moderate to severe TBI require close observation and intensive treatment from the time of injury to reduce mortality and morbidity. Clinical neurological examination and imaging criteria are used to guide interventions in awake patients with mild to moderate pTBI. More invasive monitoring is reserved for severely injured children to guide interventions using a combination of medical and surgical therapies delivered in a tiered fashion, based on the 2019 Brain Trauma Foundation guidelines.^90^

### Pre-hospital management of pTBI

The focus is on initial assessment based on Glasgow Coma Scale and pupillary examination, treatment with airway support and stabilization as needed, and triage to a paediatric trauma centre. In the most severely injured children, evidence supports aggressive prevention and treatment of hypoxia and hypotension^126,127^ and suggests improved outcomes from guideline implementation.^128^ A recent systematic review identified 10 clinical practice guidelines for pre-hospital management of pTBI, four of which were considered high quality.^129^ Among these, half of the recommendations were based on robust evidence, whereas 20% were based on expert consensus.

### Hospital management of pTBI

In the hospital phase, the management of mild pTBI focuses on assessing the imaging requirement and providing a period of observation. This is followed by a prompt, stepwise return to cognitive and physical activity, robust discharge advice and, if required, neurorehabilitation for ongoing symptoms.^57^ For children with recalcitrant moderate to severe post-concussive symptoms in the acute setting, some evidence suggests that hypertonic saline might be useful in acute management. There is limited evidence to support use of any other agents, such as methylphenidate, sertraline or amitriptyline, for similar symptoms.^130-132^ It is important to note that the vast majority of patients with mild pTBI are not managed in the hospital setting. However, evidence-based approaches for community-based care are lacking, with the exception of exercise interventions (Supplementary Box 2). Furthermore, most guidelines reflect resources relevant to HICs, which would be limited in LMIC settings.^2,133-136^

The management of moderate to severe pTBI is guided by early identification of intracranial pathology to identify the requirement for neuromonitoring, neuroprotection and medical and surgical interventions.^137^ An early mainstay of treatment is maintaining ICP and cerebral perfusion pressure at targets defined by the Brain Trauma Foundation guidelines.^4^ These are achieved by baseline neuroprotective intensive care (cardiorespiratory support to maintain normal carbon dioxide and oxygen levels, adequate blood pressure to perfuse the brain, and appropriate sedation to reduce ICP). In patients who fail to respond to these measures, increasing intensity of treatment (hyperosmolar therapy, deeper sedation, hypothermia, CSF diversion, decompression and barbiturate coma) is offered. However, there is emerging evidence questioning the utility of invasive monitoring,^138^ fixed ICP and cerebral perfusion pressure targets,^91,139,140^ in addition to therapeutic interventions such as CSF diversion^141^ and decompressive craniectomy.^142^ The only multicentre, multinational comparative effectiveness trial in severe pTBI to date, the ADAPT trial, generated important but limited data on the use of hyperosmolar therapy and CSF diversion.^141,143^ Despite these contributions, the evidence base for clinical care recommendations remains weak; consequently, clinical practice varies widely.^144^ The present treatment thresholds for ICP and cerebral perfusion pressure are inadequate for the paediatric age range.^140^ Although advanced neuromonitoring tools could help to individualize treatment targets, progress in the field has been slow.^145^ Compared with the literature on adult TBI, large-scale studies on pTBI are rare. There is an urgent need to identify age-appropriate treatment thresholds to improve outcomes for the most severely injured children.

#### Rehabilitation and post-acute care

Rehabilitation is a vital component of recovery after pTBI, with evidence of positive effects of both clinician-directed and clinician-guided parent rehabilitation (rehabilitation delivered by parent with guidance from clinician).^146-148^ Although any pTBI can lead to deficits, more severe forms of injury are more likely to affect multiple domains, including physical, cognitive, emotional, social and developmental functions. A recent systematic review identified persistent symptoms in nearly one-third of patients after sports-related concussion that lasted beyond the first 2 weeks and could benefit from cervico-vestibular rehabilitation.^149^ For children not requiring hospitalization, a return to activity and school counselling can be performed by a variety of experts, including primary care physicians, athletic trainers and school nurses, working collaboratively with families. In preparation for transition home, the focus is to identify functional difficulties that will require ongoing rehabilitation, in partnership with the school and family to support the needs of the child. For optimal benefit, regular communication and monitoring should be provided throughout this process.^2,150^

For hospitalized children, inpatient rehabilitation should be provided by a multidisciplinary team, including neuropsychology, physical therapy, occupational therapy and speech therapy, until the child is ready for discharge. Assessing and understanding communication and motor skills are paramount for an effective rehabilitation programme. Thorough cognitive assessment by trained paediatric neurorehabilitation professionals, encompassing attention, memory, learning, executive skills, behaviour regulation and social cognition, is crucial to define the ongoing neurorehabilitation requirements.^151^ Screening for mental health problems is also crucial, given its association with affective and behavioural disorders.^152^ After discharge from the hospital, children require ongoing multidisciplinary rehabilitation, either at a specialist facility or as an outpatient, depending upon the neurological deficits. This is followed by ongoing help and support at home and school. Family and social support structure is crucial for transition home.^153^

Evidence also supports use of telerehabilitation and interventions to empower parents by enhancing their skills to help reduce parental stress and improve outcomes.^154-156^ A partnership model works best, whereby clinicians, families and the child work together to identify functional difficulties requiring ongoing rehabilitation to achieve common goals.^5,157^ For continued improvement, ongoing rehabilitation and targeted interventions are warranted across the lifespan for pTBI survivors.^144,158-160^ However, rehabilitation services are insufficient in many HICs, especially for those without universal healthcare, and in the majority of LMICs.^151^ The World Health Organization (WHO) provides a rehabilitation package that can be implemented in the systems that lack proper resources.^152^

### Systems of care

Key takeaway: improving pTBI outcomes requires specialized, coordinated, interdisciplinary care across all trauma phases. Gaps in paediatric expertise persist even in HICs, and LMICs face added challenges from fragmented pre-hospital systems, limited access to specialized tertiary care and inadequate long-term rehabilitation frameworks.

**Figure 5:**
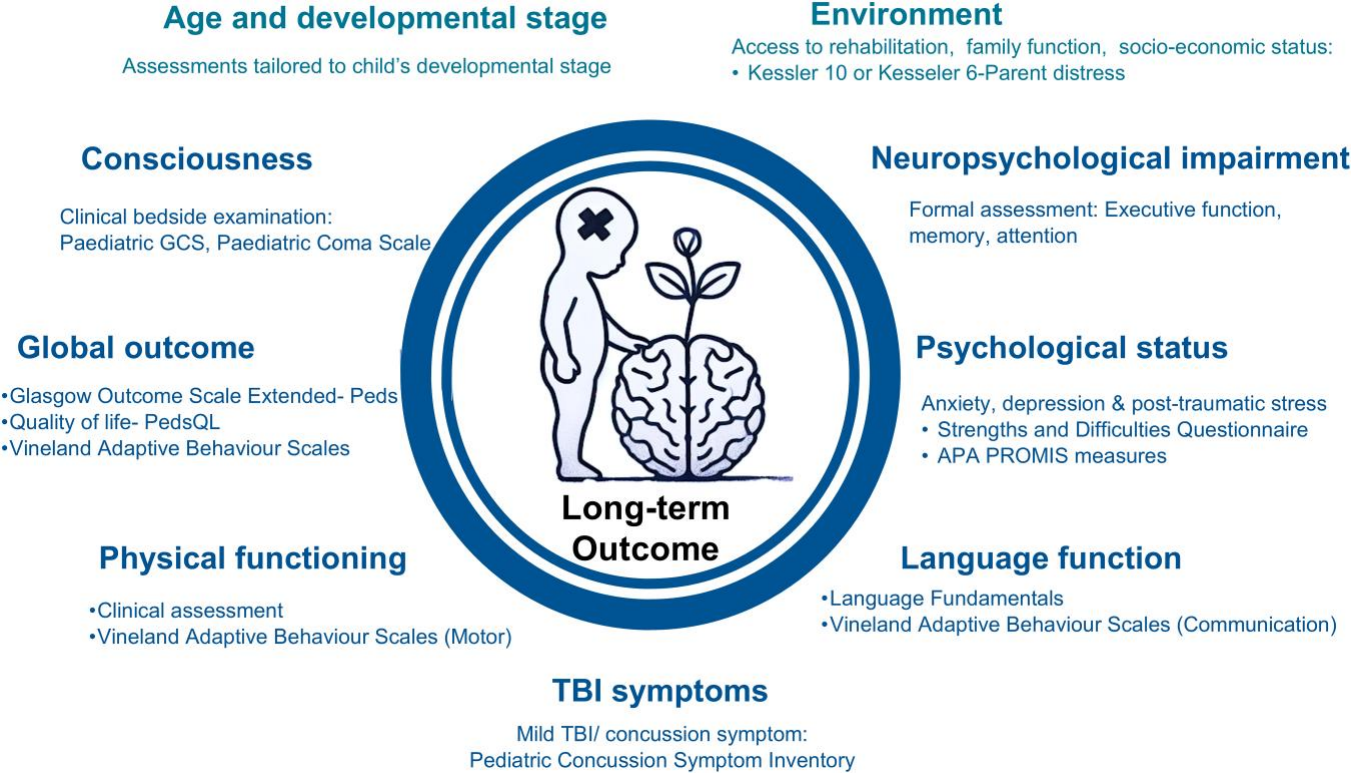
**Outcome assessment in paediatric traumatic brain injury.** Domains for evaluating long-term outcomes, with examples of commonly used standardized clinical assessment tools: Paediatric Coma Scale,^80^ Glasgow Outcome Scale Extended for Pediatrics,^81^ Quality of life (PedsQL),^82^ Vineland Adaptive Behaviour Scales,^83^ Strengths and Difficulties Questionnaire,^84^ APA PROMIS measures,^85^ Language Fundamentals,^86^ Pediatric Concussion Symptom Inventory,^87^ Kessler 10 or Kesseler 6- Parent distress.^88^ GCS = Glasgow Coma Scale; TBI = traumatic brain injury.

For the best outcomes, a robust coordinated network of healthcare systems is required, spanning acute and rehabilitative care and supported by wider social stakeholders (Fig. 5). Apart from comprehensive care after pTBI, from the pre-hospital phase all the way to rehabilitation, it should also work on prevention strategies with engagement of policy-makers, stakeholders and providers. Variations in systems of care have a huge impact on outcomes from pTBI.^161^ In LMICs, poor outcomes from pTBI are likely to represent multilayered contributions from pre-hospital, acute and post-acute phase factors. The rural areas in LMICs bear the greatest burden owing to a high incidence of pTBI and lack of access to timely specialist management.^162^ Hence, improving care in LMICs requires a deeper understanding of the stakeholders, how the clinical micro-system functions within the macro-healthcare system, and how changing operations will synergize with other healthcare improvement efforts.^163^ For optimal outcomes and wider dissemination, it is imperative that the best practice recommendations are adapted to available resources. One such example is the multi-organizational consensus guideline (https://www.ntsi.co.in/downloads, accessed 6 March 2025) developed with the support of the Neurotrauma Society of India (NTSI), the Neurological Society of India (NSI) and the American Association of Physicians of Indian Origin (AAPI). Such adaptations keep the ground realities in context when designing trauma care models and implementing guidelines.

#### Pre-hospital care

Post-injury, pre-hospital care is a critical determinant of early survival. Any delays in transport, lack of trained personnel and minimal equipment exacerbate the impact of injury. Coordinated emergency medical systems in HICs ensure appropriate triage, early intervention and timely transfer to specialized trauma centres. However, there are substantial variations in pre-hospital trauma systems worldwide, including response time and interventions on site.^164^ Of note, the understanding of pre-hospital care coordination in LMICs is incomplete owing to lack of centralized systems or robust data-collection mechanisms in many geographical areas. Despite the higher pTBI-related deaths in LMICs, pre-hospital care is often limited or absent. Implementation of robust pre-hospital guidelines might improve survival for these children.^128^ Investing in low-cost, high-yield interventions, such as first responder training and development of cost-effective triage tools, can improve pre-hospital care in LMICs.

#### Acute care

Tertiary care for pTBI in paediatric and mixed trauma centres results in lower mortality compared with adult trauma centres, even after accounting for trauma volume.^165^ This is likely to be attributable to availability of cross-disciplinary, paediatric-trained trauma teams, including nurses, surgeons, emergency physicians, anaesthetists and allied health professionals. Equally important are the provision of specialist paediatric critical care, timely access to neuroimaging and neurosurgical expertise, because delays in treatment are associated with poorer outcomes.^125^ A comparative study between LMIC and HIC paediatric cohorts showed that HIC status was correlated with a shorter time to consultation, imaging and surgery, shorter hospitalization and better outcomes among severe cases, although such differences might also occur within the same region across population groups defined by economic status and even ethnicity.^166-168^ The available pTBI guidelines need to consider regions with fewer resources for invasive neuromonitoring, in addition to the role of neurosurgery in these settings.^169,170^ Practice is non-uniform even in HICs, with considerable variability in paediatric-specific training, experience and exposure among acute care professionals.^171^ Recent evidence-based quality indicators for paediatric trauma care have been published, which might help to guide trauma systems for best outcomes in pTBI.^172^

#### Post-acute care

The impact of trauma on long-term morbidity and health economic outcomes is considerable across the lifespan of a child. However, our current knowledge is based mostly on data from HICs. Much less is known about the personal and societal burden in resource-limited settings.^173^ To reduce socio-economic burden, early, intentional and ongoing rehabilitation is essential to further the gains of successful resuscitation and acute care in pTBI.^174^ Current rehabilitation approaches focus heavily on physical rehabilitation and need to include neurorehabilitation. There is encouraging emerging evidence showing improvement in online problem solving^175^ and executive functioning^176^ with early interventions. Furthermore, standards need to be established for coordination between multiple specialties and for systematic transition back to school and the community.^177^ Caregivers play a crucial role in identifying post-pTBI behavioural changes; their systematic involvement in action-planning and goal-setting can strengthen implementation and guide rehabilitation for the best outcomes.^146,178-180^

The WHO’s 2030 Rehabilitation Initiative emphasizes the need to build comprehensive rehabilitation service delivery models for equitable access to high-quality services.^152^ Investment in scalable, evidence-based models and adapting successful models to available resources are required to address disparities in systems of care. This demands multi-sector collaboration, engaging governmental and non-governmental sectors, education systems and local communities. For sustainability, it is crucial to embed pTBI care within broader health system-strengthening initiatives.

## Prognosis and outcomes

Key takeaway: accurate outcome prediction after pTBI should account for the complex interplay of injury-related, developmental, environmental and child-specific factors. This would help in optimizing recovery and supporting families in both high- and low-resource settings.

**Figure 6:**
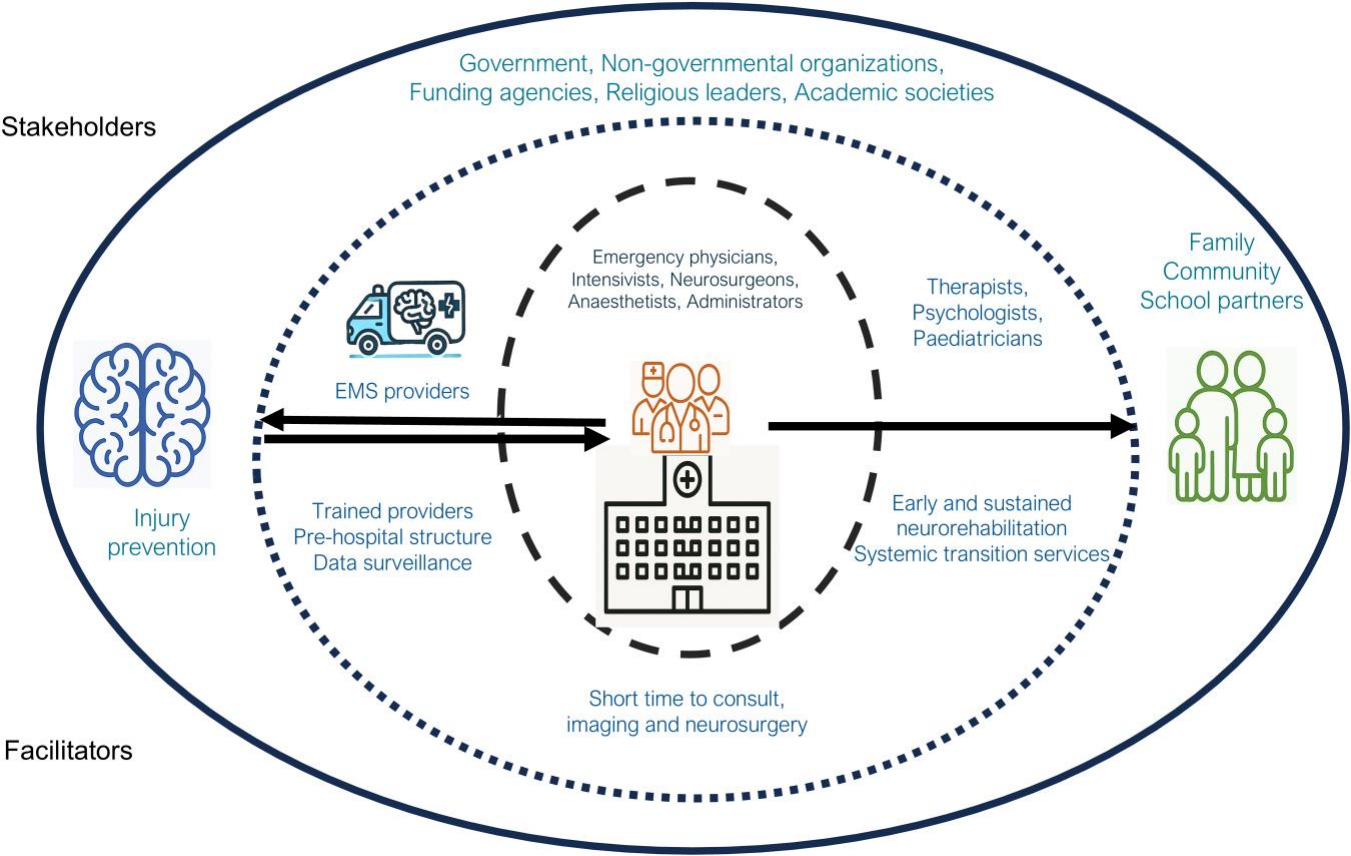
**Systems of care in paediatric traumatic brain injury.** EMS = emergency medical services; TBI = traumatic brain injury.

Accurate prognosis prediction remains elusive, owing to the wide variation in patient- and injury-related factors, outcome measures, intervention practices and research methodologies. Injury-related factors, such as the nature and severity of injury and associated complications, are critical in the acute clinical care. However, child factors also have a crucial impact on outcomes. For example, younger age is associated with generally poorer outcomes, which is likely to be a reflection of the stage of brain development at the time of injury.^43^ Likewise, pre-injury cognitive and socio-emotional status plays a role in post-injury recovery. The environment of the child, often referred to in the context of social determinants of health, influences outcomes and offers opportunities to modify recovery. Other important factors that influence outcomes can be distal (e.g. parent education or income) and/or proximal (e.g. family function, parent mental health) (Fig. 6).^181-183^ For example, the ‘double hazard’ paradigm^184^ provides a guide for predicting outcomes: severe TBI plus younger age at injury results in poorer outcome than either dimension alone, and severe TBI plus younger age at injury plus environmental disadvantage leads to even poorer outcome.

In LMICs, limited access to nutrition, healthcare, education and basic support systems (e.g. community health programmes, education initiatives and social support structures) can exacerbate the negative effects of the distal and proximal risk factors on post-pTBI recovery. Given that health priorities and resources differ across HICs and LMICs, consideration of these predictive factors might assist in directing scarce resources to the most vulnerable children. The unique practical consideration of studying the bidirectional effects of family functioning is crucial in pTBI research.^185^ Apart from challenges, the social and environmental milieu of pTBI also offers unique opportunities for cost-effective care for optimal outcomes.^154,155^ Genomic heterogeneity holds promise in explaining the unknown variability in outcomes and also potential for treatment strategies tailored to a specific genomic constitution.^108,109^

To ensure optimal support for children with persisting symptoms or deficits, best practice includes regular assessment and monitoring at key transition points through childhood and into early adulthood. Longitudinal assessments are particularly crucial in children to monitor the effect of injury on the developmental trajectory. Current clinical tools might not incorporate all the developmental variations fully, particularly in the younger age group.^60^ The optimal longitudinal assessment tools for children would ideally take account of the age/developmental stage of the child at all time points after injury, pre-injury functional status and the cultural/language background. This would ensure standardized assessments in diverse settings to capture the long-term impact of pTBI fully. Studies, such as the Predicting and Preventing Post-concussive Problems in Pediatrics [5P] study, are vital early attempts to capture and prognosticate the considerable morbidity of pTBI even in those considered ‘mild’.^186^ Future work should prioritize multicentre studies encompassing the full spectrum of pTBI severity across diverse populations and focus on developing standardized protocols that integrate crucial clinical considerations.

## Prevention of paediatric TBI

Key takeaway: prevention is the most powerful intervention in mitigating the public health burden of pTBI.

Efforts aimed at prevention represent the most cost-effective cornerstone for reducing the disease burden associated with pTBI. Broadly speaking, children are at a particularly high risk for TBI owing to falls, sports injuries, road traffic accidents and motor vehicle crashes. Effective prevention strategies involve a multi-pronged approach including public education, a child-friendly environment and legislative action. The Haddon Matrix provides a systematic framework to approach pTBI prevention efforts (Supplementary Box 3), although such approaches are dependent on resources. Therefore, a deep knowledge of the most effective and easily available local and regional policy tools or interventions is required to drive systemic change.

Effective prevention efforts begin with characterizing the basic epidemiology of pTBI, which differs markedly between LMICs and HICs. Given that the incidence and outcomes of pTBI are affected by racial, geographical and socio-economic disparities, prevention must be comprehensive and equity-informed. Rural communities, for instance, often experience delayed access to care, compounding the impact of injury. A systems-level approach (integrating healthcare providers, schools, families and policy-makers) is necessary to promote widespread and sustained prevention efforts. Additionally, improving public understanding of pTBI is essential to ensure early identification, appropriate follow-up and long-term support.

The WHO has an active ongoing campaign towards prevention of injuries through a public health initiative that can be adopted to local needs.^187^ Universal implementation of simple, cost-effective strategies, such as speed reduction, creation of pedestrian-safe zones, enforcement of child car seat and seatbelt laws and helmet use, have significant potential to reduce pTBI. Likewise, appropriately designed play areas with impact-absorbing surfaces and safer home and school environments to prevent falls can substantially reduce the fall-related incidence of pTBI. Enhancing public awareness through education and school-based programmes can improve attitudes and behaviour towards risk mitigation and injury prevention. There is substantial scope to reduce AHT around the world through wider implementation of public health interventions aimed at empowering and educating parents.^188^

## Research and best practices in paediatric traumatic brain injury

Key takeaway: high-quality evidence in pTBI is limited and creates barriers to implementing best practices, emphasizing the need for enhancing research funding to derive data-driven consensus in treatment approaches.

### Current state of research and guidelines

**Figure 7:**
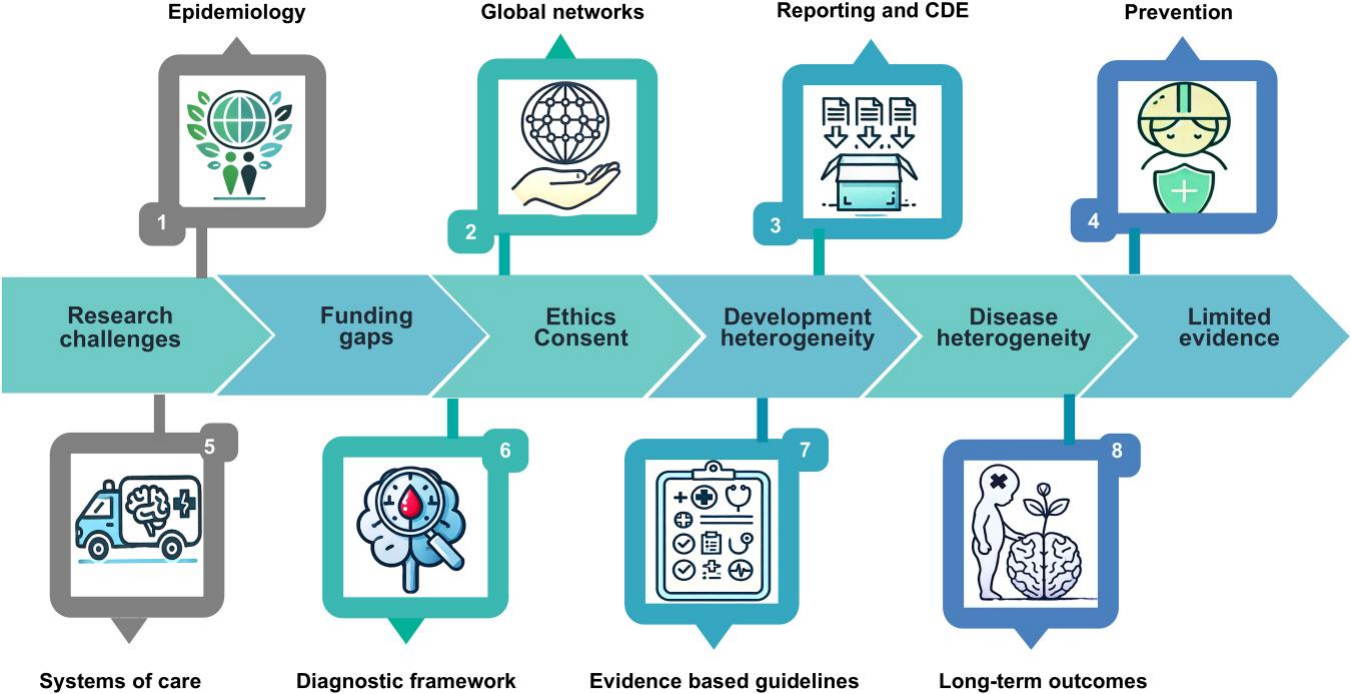
**Paediatric traumatic brain injury: unique population, unique challenges.** Challenges and priorities for pTBI highlighted across eight domains: (1) epidemiology; (2) global networks; (3) reporting and CDE; (4) prevention; (5) systems of care; (6) diagnostic framework; (7) evidence based guidelines; and (8) long-term outcomes. Details of key findings and recommendations for each domain are provided in Table 1. CDE = common data elements; TBI = traumatic brain injury.

Despite the recommendations from various guidelines for required research, there is a paucity of high-quality evidence. The paediatric age poses unique challenges whilst designing and conducting research. As outlined in previous sections, developmental differences, regional and resource-dependent differences and socio-economic disparity in individual countries bring heterogeneity and complexity to research protocols. Additionally, the ethics of research in acute illness, particularly in children, is complex owing to difficulties in the consenting process and long-term follow-ups. This limits the true understanding of the disease burden, its impact on long-term outcomes, DALY and socio-economic costs to the society. The progress in scientific research in pTBI is slow; in total, 174 studies have been registered on the International Clinical Trials Registry Platform in the past 10 years, of which 104 are interventional and 70 are observational studies. The majority of these studies are from HICs with limited extension in LMICs, although the platform might not capture all the ongoing research. Also noticeable is the relative lack of large multicentre, multinational cohort studies, which should be the aspiration for robust evidence. Any of the 47 international guidelines pertaining to pTBI, published at the writing of this manuscript, are frequently cited as insufficient to change clinical care of experienced providers. The barriers to best practices and impactful research in pTBI describe this conundrum between quality of evidence available and generation of guidelines (Fig. 7 and Table 1).

**Table 1:** Key findings, research and policy recommendation for paediatric traumatic brain injury

Domain	Key findings	Research/policy recommendations
Epidemiology	True global incidence and prevalence of pTBI are unknown, particularly in LMICs. Environmental and social factors influence which children sustain a TBI and how well they recover.	There needs to be international infrastructure, funding and agreed methods for epidemiological reporting. Studies of pTBI should include a minimum dataset that captures these variables.
Networks	Local and global clinical and research networks must be strengthened and integrate multidisciplinary expertise and lived experience. Such networks can share best practice and resource access and provide research infrastructure.	Establish local networks integrating national and international structures. Develop robust best practice guidelines tailored to local epidemiology and resources to ensure that evidence supports clinical care.
Common data	Paediatric-specific CDEs and reporting pipelines for characterizing injury, biomarkers, clinical treatment and outcome need development and refinement.	Support development and implementation of appropriate norms for CDE, clinical and biomarker variables for pTBI. Develop a pragmatic minimum dataset, which is also practicable in resource-poor environments.
Prevention	The incidence of pTBI can be reduced and/or its impact mitigated by effective prevention strategies. Environmental and social determinants should dictate the implementation of preventive strategies. Developmental considerations should be addressed to account for developmental peculiarities across the paediatric age range.	Prioritize implementation and enforcement of preventive measures universally. Support public health campaigns to raise safety awareness. Provide parental support and locally adapted educational programmes to reduce pTBI, particularly AHT. Optimize age-appropriate prevention, such as cycle helmets and child restraints in cars. Sports with high concussion risk should explore and implement evidence-based modifications in rules and age thresholds to ensure protection of developing brains.
Systems of care	Optimizing systems of care for all severities of pTBI can deliver rapid improvements in care, beyond any achievable by any single intervention.	Engage key stakeholders (particularly in LMICs) to support policy development and funds for effective implementation. Paediatric expertise should be available in caring for pTBI.
Diagnosis	There is lack of validated assessments tools for children. Biomarkers can help to identify underlying pathobiology, reduce radiation exposure, and provide early detection. Developmental considerations are paramount in establishing age-appropriate biomarkers before their use can be implemented in clinical practice.	Age-appropriate norms and thresholds need to be defined urgently for available targets. Age-appropriate biomarker assays need to be identified to facilitate use in younger children. Neuro-imaging biomarkers and validated scoring methods should consider developmental changes. Feasibility of ultrafast MRI, refinement and validation should be tested in pTBI to reduce radiation risks.
Guidelines	Adherence to guidelines and early, tiered treatment tailored to injury severity of injury can improve outcome, but supporting evidence is limited. Environmental and social determinants should dictate guidelines for effective implementation. Developmental considerations are lacking in current guidelines owing to lack of available scientific evidence.	Evidence-based guidelines should be created both for resource-rich and poor settings and for hospitalized and non-hospitalized children following TBI. Research-informed pTBI management guidelines require global networking, funding and engagement of stakeholders. Non-invasive neuro-monitoring tools should be validated for use in resource-poor environments. There is urgent need to define age-appropriate thresholds.
Long-term outcome	Accurate prediction is essential to tailor appropriate rehabilitation for transition to home, school and society. Environmental and social determinants dictate outcome and need to be considered in all pTBI outcomes. Developmental trajectory is important in assessing the real impact of TBI in a child.	Implement legislation and policy to enable linkage studies to capture long-term follow-up. Urgent funding and policy are needed to develop paediatric rehabilitation services, in both LMICs and HICs. A gap assessment is needed for post-pTBI mental health and work with juvenile offenders and young adult prisons. Age-appropriate prediction models need to be developed for robust assessment of neurological outcome.

AHT = abusive head trauma; CDE = common data element; HICs = high-income countries; LMICs = low- and middle-income countries; pTBI = paediatric traumatic brain injury.

### Future directions and examples of good practice

For evidence-informed practice in pTBI, large multicentre international collaborative efforts and innovative research designs are needed. This requires appropriate funding, regulatory approvals and engagement of policy-makers and healthcare professionals alike. Despite the highlighted challenges above, there are encouraging advances in pTBI research, largely driven by collaborative efforts and expert working groups. International collaboration to create shared data repositories and multicentre studies have been pivotal in synthesizing existing consensus statements and identifying research priorities. Building on this foundation, future work should focus on expansion of these to address existing gaps and strengthen the pTBI-specific knowledge base.

#### Collaborative networks

There are increasing numbers of successful collaborative research networks for pTBI research. PACCMAN (Pediatric Acute & Critical Care Medicine Asian Network, 50 paediatric intensive care units from Asia) and LARed (Red Colaborativa Pediátrica de Latinoamérica, 30 paediatric intensive care units from Latin America) have created an international research network covering many LMICs. In the past 5 years, the collaboration has pooled data on available resources across different centres for pre-hospital and emergency management,^189^ clinical outcomes from pTBI,^161^ and paediatric intensive care unit outcomes.^190,191^ The group have conducted prospective research to answer management questions, for example, regarding a better osmolar agent for improved outcomes from pTBI.^192^ Likewise, ENGIMA (Enhancing NeuroImaging Genetics through Meta-Analysis) is a global collaborative to advance research through meta-analysis of neuroimaging in pTBI.^116,193^

The collective experience from successful national networks (predominantly in HICs) has benefitted all healthcare settings globally. For example, the decision tools for neuroimaging in mild TBI created by several national emergency medicine societieshave introduced objective criteria to standardize imaging practices and minimize unnecessary radiation exposure.^64-68^ Likewise, the CTRC (Canadian Traumatic Brain Injury Consortium) has several active research projects in the pTBI domain ranging from mild to severe TBI, utility of biomarkers, acute management and rehabilitation.^194^ The Pediatric Brain Injury Consortium in the USA^195^ is a network of rehabilitation experts to establish common data elements for further research in pTBI. An ideal expansion of such networks would include LMICs to enable collection of global epidemiological data whilst supporting local adaptation of available resources in diverse settings. Experience from the Global Neuro Trauma Network for adult TBI (https://globalneuro.org/EN/about/about-global-neuro.html)^196^ could be used to create a similar initiative in pTBI or potentially be expanded to include children. Likewise, existing paediatric networks could be expanded to disseminate existing pTBI knowledge and facilitate implementation of management guidelines across all healthcare set-ups. Learning from the success of large adult TBI collaborations, such as TRACK-TBI and CENTER-TBI, pooling resources and expertise from existing paediatric networks would also be a cost-effective strategy to establish the critical evidence base required to inform pTBI management.^197,198^

#### Innovative study designs

Comparative effectiveness studies have been hugely successful in creating an evidence base for adult TBI.^197,198^ A similar pTBI study, the ADAPT Trial, has created a research database of 1000 severe pTBI patients recruited from several international sites. It is hoped that continued analyses of ADAPT and similar pTBI databases using innovative data science approaches will help to delineate best practices and lead to future advances. Incorporating real-world data and machine-learning techniques could help in learning from routinely collected clinical data.^91^ Similar to the IMPACT collaboration, the next step for pTBI research could be to combine existing pTBI data from prospective and retrospective studies.^199^ Given the difficulties in conducting research in children, this would be a useful step to identify paediatric-specific prognostic models, conduct age-stratified analysis and help to define the common data elements for pTBI research. Using a telemedicine approach to collect long-term outcome data and implement virtual interventions through caregivers could prove immensely useful in improving research uptake.^156^ It is equally important to explore the full impact of interplay between injury factors and proximal factors on long-term outcomes, especially the family burden, mental health and socio-economic background. Given that sub-acute follow-up studies are more challenging than acute studies, data linkage studies and information sharing through a collaborative network approach could help to address these gaps.

## Conclusion

Paediatric TBI is a significant global health burden and demands a multifaceted, child-centred response. This review highlights the unique vulnerabilities of the developing brain, wide global disparities in care and lack of paediatric-specific evidence. The eight key domains requiring focused attention (epidemiology; pTBI networks; reporting and common data elements to strengthen pTBI-specific evidence; pTBI prevention; systems of care; diagnostic framework; evidence-based guidelines; and long-term outcomes) are presented in Fig. 7, with detailed recommendations for each domain summarized in Table 1.

Targeted interventions, when supported by policy, education and equitable access to safety infrastructure, can significantly reduce the incidence and impact of pTBI. A systems-level approach integrating public health, education and healthcare is crucial for injury prevention and safeguarding neurodevelopmental health across populations. Robust comprehensive evidence-based guidelines for diagnosis and management, and long-term neurorehabilitation have the potential to improve outcomes.

Bridging gaps across the continuum of care, reducing disparities and implementing scalable models are crucial for improving the quality, accessibility and equity of pTBI care globally. Sustainable progress requires increased investment in prevention and research infrastructure, with active engagement of appropriate stakeholders to foster innovation and strengthen cross-sector partnerships to expand existing networks.

## Supplementary Material

awaf459_Supplementary_Data
