# Supplementary material for: Paediatric traumatic brain injury: unique population and unique challenges
Source: Brain. 2025 Dec 13;149(3):736–56. doi: 10.1093/brain/awaf459 (PMC13016995; doi:10.1093/brain/awaf459)
Supplement: awaf459_Supplementary_Data [file awaf459_supplementary_data.pdf]

Supplemental Methods

Search Strategy: This narrative review was performed in accordance with the RAMESES (Realist And Meta-narrative Evidence Syntheses – Evolving Standards) project. Initial searches were run in PubMed to identify keywords and indexing terms. A medical librarian then combined these terms logically using Boolean logic to create a sensitive search across the following concepts: traumatic brain injury, pediatrics. All searches were run on the same date (March 2025) and results were deduplicated in EndNote following the validated deduplication method put forth by Bramer et al. Results were imported into Covidence systematic review software. Updates were run twice during the manuscript drafting, and Covidence was used to deduplicate these results. Titles and abstracts were independently reviewed by RM against predetermined eligibility criteria. Full text PDFs of eligible citations were then reviewed via the same process.

((((pediatrics[MeSH] OR infant[MeSH] OR "infant, newborn"[MeSH] OR "infant, low birth weight"[MeSH] OR "infant, premature"[MeSH] OR child[MeSH] OR adolescent[MeSH] OR pediatric*[Title/Abstract] OR paediatric*[Title/Abstract] OR baby[Title/Abstract] OR babies[Title/Abstract] OR infant*[Title/Abstract] OR neonat*[Title/Abstract] OR newborn*[Title/Abstract] OR toddler*[Title/Abstract] OR child[Title/Abstract] OR children[Title/Abstract] OR childhood[Title/Abstract] OR adolescen*[Title/Abstract] OR teen*[Title/Abstract] OR youth*[Title/Abstract] OR "young people*"[Title/Abstract]) AND
(("head injuries, closed"[MeSH] OR "brain hemorrhage, traumatic"[MeSH] OR "brain injuries, traumatic"[MeSH] OR "brain contusion"[MeSH] OR "shaken baby syndrome"[MeSH] OR "head injuries, penetrating"[MeSH] OR "intracranial hemorrhage, traumatic"[MeSH] OR concussion*[Title/Abstract] OR concussive*[Title/Abstract] OR "head trauma"[Title/Abstract] OR "head injur*"[Title/Abstract] OR TBI[Title/Abstract] OR TBIs[Title/Abstract] OR "shaken baby syndrome*"[Title/Abstract]) OR
((brain[MeSH] OR brain*[Title/Abstract] OR cerebral[Title/Abstract] OR craniocerebral[Title/Abstract] OR encephalon*[Title/Abstract] OR "gray matter"[Title/Abstract] OR intracranial*[Title/Abstract] OR "white matter"[Title/Abstract])
AND
("wounds and injuries"[MeSH:noexp] OR hemorrhage[MeSH:noexp] OR "intracranial hemorrhages"[MeSH] OR "cerebral hemorrhage"[MeSH] OR hematoma[MeSH:noexp] OR "hematoma, epidural, cranial"[MeSH] OR "hematoma, subdural"[MeSH] OR "brain injuries"[MeSH] OR "brain injuries, diffuse"[MeSH] OR injur*[Title/Abstract] OR wound*[Title/Abstract] OR bleed*[Title/Abstract] OR haemorrhage*[Title/Abstract] OR hemorrhage*[Title/Abstract] OR bruise*[Title/Abstract] OR contusion*[Title/Abstract] OR hematoma*[Title/Abstract]) AND
("wounds, nonpenetrating"[MeSH] OR "wounds, penetrating"[MeSH] OR "wounds, gunshot"[MeSH] OR "wounds, stab"[MeSH] OR "accidental falls"[MeSH] OR "accidents, traffic"[MeSH] OR "athletic injuries"[MeSH] OR trauma*[Title/Abstract] OR "blunt injur*"[Title/Abstract] OR "blunt wound*"[Title/Abstract] OR "nonpenetrating injur*"[Title/Abstract] OR "nonpenetrating wound*"[Title/Abstract] OR "penetrating injur*"[Title/Abstract] OR "penetrating wound*"[Title/Abstract] OR gun[Title/Abstract] OR guns[Title/Abstract] OR gunshot*[Title/Abstract] OR firearm*[Title/Abstract] OR "fire arm*"[Title/Abstract] OR stab[Title/Abstract]

OR stabs[Title/Abstract] OR stabbed[Title/Abstract] OR stabbing[Title/Abstract] OR fall[Title/Abstract] OR falls[Title/Abstract] OR falling[Title/Abstract] OR "traffic accident*"[Title/Abstract] OR "traffic collision*"[Title/Abstract] OR "car accident*"[Title/Abstract] OR "car collision*"[Title/Abstract] OR "motor vehicle accident*"[Title/Abstract] OR "motor vehicle collision*"[Title/Abstract] OR "athletic injur*"[Title/Abstract] OR "sports injur*"[Title/Abstract])))) NOT ("case reports"[Publication Type] OR "case stud*"[Title] OR "case report*"[Title] OR “case series*”[Title]))NOT (animal[Filter] NOT humans[Filter])) AND (y_5[Filter])

Supplemental Box 1: Abusive Head Trauma (AHT)

Definition and Mechanism:
Previously known as "shaken baby syndrome", AHT involves shaking and/or blunt impact. It is the leading cause of fatal traumatic brain injury (TBI) in children under two years of age. Injuries are caused by acceleration-deceleration forces that result in subdural haematoma, retinal haemorrhages, and skull or rib fractures.

Risk Factors:
Risk factors include infant crying, young or inexperienced caregivers, single-parent households, and low socioeconomic status. Perpetrators are often fathers, stepfathers, or male caregivers, but mothers and female babysitters may also be responsible.

Diagnosis:
Diagnosis requires a careful history, physical examination, and radiological imaging. Key indicators include subdural haematoma, retinal haemorrhages, and unexplained injuries inconsistent with given history.

Treatment:
Treatment involves supportive care, maintaining vital signs, controlling intracranial pressure, and surgical intervention if needed. Seizures and intracranial hypertension are common complications, requiring close monitoring and management.

Outcomes:
Survivors may suffer from lifelong cognitive, motor, and sensory disabilities, including blindness, seizures, and developmental delays.

Prevention:
Public education programs, such as the ‘Period of PURPLE Crying’, are critical for prevention (https://dontshake.org/purple-crying). Healthcare providers play a key role in early recognition and intervention.

Supplemental Box 2: Mild Paediatric Traumatic Brain Injury Management

**Education and Reassurance**	Healthcare professionals should provide comprehensive education to families, including:<br>- warning signs of severe injury,<br>- expected symptom course,<br>- monitoring instructions,<br>- cognitive and physical activity management,<br>- return-to-school and recreational activity guidelines.
**Rest and Gradual Return to Activity**	Initial physical and cognitive rest for no more than 1-2 days post-injury (moderate; level B)<br>Gradual return to activity that does not worsen symptoms (moderate; level B)<br>Active rehabilitation with non-contact aerobic activities once initial recovery is underway (high; level B)
**Return to School**	Customized return-to-school protocols based on symptom severity created collaboratively by medical and school teams (moderate; level B)<br>Monitoring and adjustments should be ongoing until the child returns to pre-injury academic performance (moderate; level B)
**Symptom Management**	Headache management includes nonopioid analgesics with caution to prevent overuse (high; level B)<br>Persistent symptoms may require multidisciplinary evaluation (moderate; level B)
**Vestibular and Cognitive Treatment**	Referral for vestibular rehabilitation may be considered for persistent vestibulo-ocular dysfunction (moderate; level C)<br>Cognitive issues should be evaluated within the context of overall symptoms, with treatment tailored to the underlying causes (moderate; level B)

Reference: *Lumba-Brown A, Yeates KO, Sarmiento K, et al. Centers for Disease Control and Prevention Guideline on the Diagnosis and Management of Mild Traumatic Brain Injury Among Children. JAMA Pediatr. 2018 Nov 1;172(11):e182853. doi: 10.1001/jamapediatrics.2018.285*

Supplementary Box 3: Haddon's Matrix for Paediatric Traumatic Brain Injury

Phase	Host	Equipment	Environment	
			Physical	Social
Pre-Event	Paediatric TBI Knowledge/training<br><br>Susceptibility/resilience for TBI<br>Supervision	Absence of Road safety<br>Absence of Safety gear (e.g. seatbelts, booster seats)<br>Absence of Protective gear (e.g. helmets, pads)<br>Absence of Playground Safety	Urban Planning<br>Access to safety gear<br>Access to protective gear	Knowledge of epidemiology<br><br>Paediatric TBI Education<br><br>Safety/protective gear perception<br>Costs of safety/protective gear<br><br>Enforcement of Rules and laws
Event	Adherence/access to safety/protective gear<br>Physical Size/Age related vulnerability	Force of impact<br>Safety & protective gear	Built environment<br><br>Safety/protective gear<br><br>Detection of TBI	Enforcement of rules and laws<br><br>Safety/protective gear enforcement
Post-Event	Symptom reporting<br><br>Ability to enact "best practices"<br><br>Susceptibility/resilience factors<br><br>Paediatric physiology	Paediatric protocols/equipment<br><br>Access to care<br><br>Hospital paediatric readiness	Access to a hospital with paediatric expertise<br><br>"Best practices" in hospital and community TBI care	Paediatric trained workforce<br><br>Neurosurgical expertise<br><br>Expense/cost of medical system<br><br>Social support<br><br>Community response to TBI
